# Mixed fermentation and electrospray drying for the development of a novel stabilized wheat germ powder containing highly viable probiotic cultures

**DOI:** 10.1002/fsn3.3092

**Published:** 2023-01-11

**Authors:** Ehsan Divan Khosroshahi, Seyed Hadi Razavi, Hossein Kiani, Ali Aghakhani

**Affiliations:** ^1^ Bioprocess Engineering Laboratory (BPEL) Department of Food Science and Engineering, Faculty of Agricultural Engineering and Technology, University of Tehran Karaj Iran; ^2^ Bioprocessing and Biodetection Lab (BBL) Department of Food Science and Engineering, Faculty of Agricultural Engineering and Technology, University of Tehran Karaj Iran

**Keywords:** electrospray, mixed fermentation, probiotic powder, shelf‐life, viability, wheat germ

## Abstract

Nondairy fermented probiotic powder was developed based on stabilized wheat germ through mixed fermentation (*Lactobacillus acidophilus* and *Lactobacillus plantarum*) and electrospraying process. In the first step, the effect of mixed fermentation on lipase and lipoxygenase activity of wheat germ was investigated. The results showed a significant reduction in the activity of both enzymes (82.72% for lipase and 72% for lipoxygenase), therefore, mixed fermentation effectively stabilizes the wheat germ. In the next step, after the preparation of the solutions for drying process and investigating the physical properties (surface tension, electrical conductivity, and viscosity) of the solutions**,** the electrosprayability of the samples was evaluated at different conditions and revealed that 18 kV applying voltage, 0.3 flow rate, and 12 cm distance between tip to collector was the best for electrospraying the 20% solution of fermented wheat germ with morphologically most semi‐uniform particles. Finally, the viability of the probiotics after drying process and during the storage at 25°C was examined. The number of initial cells counted as 14.48 ± 0.2 log cfu/g and the viability studies showed 0.55 log cfu/g decrease in the number of viable bacteria from initial count as a result of the electrospraying process. Furthermore, 7.86 ± 0.03 log cfu/g in freeze‐dried and 9.05 ± 0.45 log cfu/g in electrosprayed samples survived after 70 days of storage.

## Graphical abstract

Wheat germ is a potential source of prebiotics though exhibiting an unstable nature; Mixed fermentation reduces the lipase and lipoxygenase activity of wheat germ; Fermented wheat germ solution has good electrosprayability; More than 96% of probiotics maintained their viability in electrospraying process; Electrospraying does less harm to the bacteria in comparison with freeze‐drying;

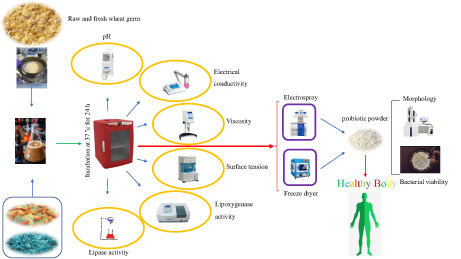

## 1 INTRODUCTION

Growing market of probiotic products due to the new lifestyle of people and increasing their awareness about nutritional and health beneficial effects of probiotics persuade many researchers to formulate new probiotic products with extended shelf‐life (Chavan et al., 2018; Companys et al., 2020; Ilango & Antony, 2021; Kumar et al., 2022; Zendeboodi et al., 2020). Among these products, according to the people who are allergic to milk protein and having lactose intolerance, nondairy probiotic products can be used by a wider community (De Bellis et al., 2021; Ilango & Antony, 2021; Küçükgöz & Trząskowska, 2022; Kumar et al., 2022). On the other hand, these days, the valorization and utilization of by‐products from different food industries are of immense importance (Calvano & Tamborrino, 2022; Castro‐Muñoz et al., 2022; Chamorro et al., 2022). Wheat germ (WG), a chief nutritious by‐product of the wheat milling process can be a potential source of prebiotic compound though exhibiting an unstable essence (Boukid et al., 2018). Therefore, applying a proper technique to the development of a novel stabilized WG powder containing highly viable probiotic cultures is economically and nutritionally important.

Probiotics are “live micro‐organisms which, when administered in adequate amounts, confer a health benefit on the host” (FAO/WHO, 2006). Prevention and treatment of gastrointestinal tract diseases (irritable bowel syndrome), prevention and therapy of allergies, definite anticancer potentials, and immunomodulation are among the health benefits (Wang et al., 2022). In recent years, *Lactobacillus acidophilus* and *Lactobacillus plantarum* have been two of the most widely used probiotic strains to develop new functional foods (Poletto et al., 2019). Keeping probiotics alive during the storage and consumption is vital to exert their therapeutic efficacy (Frakolaki et al., 2020; Sirini et al., 2021). At present, most of the probiotic products are in liquid refrigerated form based on fermented dairy substrates that require temperatures below 4°C. Providing this temperature during the maintenance and transportation period is costly, though development of a probiotic powder with extended shelf‐life at ambient temperature facilitates the portability conditions, promotes usability in various food products (Librán et al., 2017), and reduces the costs (Ben, 2016; Moumita et al., 2018). For this purpose, different drying encapsulation methods such as freeze‐drying (Moayyedi et al., 2018), spray‐drying (Moreno et al., 2022; dos Santos et al., 2019), and electrospraying have been applied. Among these, electrospraying is known as a new and efficient method that works under a mild condition (single stage, atmospheric environment, ambient temperature, lower energy consumption, and avoiding the use of organic solvents) (Castro Coelho et al., 2021). In this method, atomization of the liquid and the solvent evaporation caused by a high voltage is applied to a viscose polymer solution between two electrodes. When the applied electric field overcomes the surface tension of the solution, a charged jet is ejected toward the collector forming fine highly charged droplets commonly shaped in spherical morphologies (Castro Coelho et al., 2021; Librán et al., 2017; Moreno et al., 2022).

WG, a main by‐product of the wheat milling industry, provides concentrated nutrients of biological components such as essential amino acids, unsaturated fatty acids, minerals, vitamins (B and E), dietary fiber, and phytosterols (Jamdar et al., 2021; Liaqat et al., 2021; Mohammadi et al., 2021; Starzyńska‐Janiszewska et al., 2021; Zhao et al., 2021), thus can be a potential candidate for prebiotics (Boukid et al., 2018). Live probiotics along with prebiotics have been suggested with innumerable health beneficial potentials (Rashidinejad et al., 2020). Approximately 25 million tons of WG are produced annually throughout the world (Kose & Yagmur., 2022), but an unstable nature due to the activity of endogenous enzymes especially lipase and lipoxygenase cause quick rancidity, hence currently, large quantities of WG worldwide are consumed just as animal feed, and its human consumption is very limited (Boukid et al., 2018). Therefore, prolonging the shelf‐life of WG is necessary (Gili et al., 2017; Tolouie et al., 2018). To reduce the enzymes activity and stabilizing the WG, many different methods such as physical, chemical, and biological have been evaluated. However, fermentation is still one of the most effective and safe methods (Boukid et al., 2018). The potential of microbial enzymes or fermentation to process food products for different purposes has long been proven, and the need for its optimal use in modern biotechnology is becoming more and more important (Kårlund et al., 2020; Valero‐Cases et al., 2020; Wuyts et al., 2020). Lactic acid fermentation is among the widely applied in food industry and is known as a promising tool for improving the shelf‐life and health characteristics of food products (Kavitake et al., 2018; Ye et al., 2019). WG fermentation by lactic acid bacteria could decrease the lipase and lipoxygenase activity and expand the shelf‐life of WG (Boukid et al., 2018; Khosroshahi et al., 2022).

Therefore, the purpose of this article was to investigate the effect of mixed lactic acid fermentation on WG stabilization, examination of the physical properties (surface tension, conductivity, and viscosity), and electrosprayability of fermented wheat germ (FWG) containing probiotic cultures, and formulate a novel nondairy probiotic product in powder form with an extended shelf‐life. Produced powders were morphologically characterized and the survivability of the probiotics during the drying process and also the viability during the storage for 70 days at 25°C were evaluated.

## 2 MATERIALS AND METHODS

### 2.1 Materials

The crude and fresh WG was obtained from Varamin flour, Co. Prior to use for fermentation trials and analysis, in order to attenuate enzymatic activity and rancidity, the samples were stored at −18°C in a two‐layer package of aluminum and polyethylene with obstacles against light and oxygen. Pure olive oil was purchased from a local market. Tween 20 as a nonionic surfactant was supplied from Sigma Chemical, Co. Culture media (MRS agar and MRS broth) were obtained by Merck, Germany and all other chemical compounds and reagents were of analytical grade.

### 2.2 Bacterial strains and inoculum preparation

*Lactobacillus acidophilus and L. plantarum* as probiotic strains were supplied from the culture collection of Bioprocess Engineering Laboratory, Department of Food Science and Engineering, University of Tehran, Iran and activated with two successful passage in sterilized MRS (Man Rogosa and Sharpe) broth from Merck at 37°C for 12 h. Then, 40% (v/v) sterilized glycerol was added to the culture and stored at −20°C in sterile screw‐cap tubes. After activation, to get the exact required cell count (10^9^ cfu/ml), the growth of *L. acidophilus* and *L. plantarum* was assessed individually in the MRS broth and simultaneously spectrophotometer was applied for optical density assessment at the absorbance of 600 nm. According to the growth kinetics of bacteria, it was revealed that *L. acidophilus* in OD = 1.68 and *L. plantarum* in OD = 1.528 reach to about 9.0 log cfu/ml.

For inoculum preparation, the stock culture of bacteria was inoculated in 10 ml of MRS broth at 37°C until a cell concentration of 9.0 log cfu/ml. Then, to get the pure cell pellets, the method described by Dias et al. (2018) was used. The centrifugation of activated culture was then conducted in sterilized centrifuge bottles at 4307 *g* for 15 min at 4°C using a benchtop refrigerated centrifuge. In the next step, supernatant portion was decanted and microbial pellets were washed in sterilized bottle using sterilized saline solution and centrifuged. Finally, the washed pellets were recentrifuged at 4307 *g* for 15 min at 4°C to remove traces of MRS broth.

### 2.3 Suspension preparation of WG

WG flakes were well grinded and sieved with mesh no. 50, then for suspensions containing 5%, 10%, 15%, and 20% WG, 5, 10, 15, and 20 g of WG, respectively, were mixed with 100 ml distilled water and they were then pasteurized at 85°C for 15 min.

### 2.4 Fermentation process

The initial volume of the inoculum was 1% v/v, thus, cocultures of 0.5% v/v *L. acidophilus* and 0.5% v/v *L. plantarum* were inoculated in every sample and were shaken well and incubated at 37°C for 24 h in an incubator (Binder Industry, Co.,).

### 2.5 pH

The pH was evaluated using a digital pH meter calibrated with buffers at pH 4.0 and 7.0. The measurements were performed with three replications.

### 2.6 Lipase activity

Lipase activity was assessed according to the method described by Kumar et al. (2013) with some modification, and here pure olive oil was replaced by triacetin as substrate. WG (500 mg) was dispersed in 15 ml of distilled water with sodium phosphate buffer adjusted to pH = 8. The free fatty acids result from the enzyme activity in 30 min, were specified by titrating against a standard solution of 0.05 M NaOH, and using of phenolphthalein as an indicator of the endpoint of titration. A blank sample was prepared for every suspension in order to investigate the changes in acidity of the suspension of WG only, without the addition of the pure olive oil as enzyme substrate. All experiments were carried out in triplicate and the lipase activity specified as milliliter of NaOH consumed at 30 min of reaction.

### 2.7 Lipoxygenase activity

Evaluation of lipoxygenase activity was conducted spectrophotometrically at 234 nm by monitoring the hydroperoxidation of linoleic acid according to the procedure described previously (Xu et al., 2013).

#### 2.7.1 Enzyme extraction

One gram of WG was mixed with 5 ml of 0.1 M cold potassium phosphate buffer (pH 6.0) and was extracted by shaking well for 1 h at 4°C. The centrifugation of slurry was then carried out at 11,000 *g* for 15 min at 4°C. Finally, after filtration of the prepared solution through a 0.20 μm filter, the filtrate was used as the source of lipoxygenase.

#### 2.7.2 Preparation of substrate solution

The substrate solution was prepared by dissolving 0.5 ml of linoleic acid (≥99%) drop by drop to the mixture of 0.5 ml of Tween 20 and 10 ml of 0.1 M borate buffer at pH 9.0. In the next step, clarification of the solution was performed by adding 1.3 ml of 1 *N* NaOH and well shaking for few minutes. Finally, by addition of 90 ml of the borate buffer, the solution was made up to a final volume of 200 ml using distilled water.

#### 2.7.3 Enzyme and substrate reaction

The mixture of 40 μl of the substrate with 2.9 ml 0.1 M potassium phosphate buffer (pH 6.0) was made and 40 μl of the WG filtrate was added as an enzyme source. Then, the alteration in absorbance of the mixture at 234 nm for 5 min was compared with the absorbance of control samples and the lipoxygenase activity was determined as follows.

$$X\left(\%\right)=\frac{\text{ΔAbs}234\;\mathrm{nm}/\text{mincontrol}}{\text{ΔAbs}234\;\mathrm{nm}/\text{mincontrol}}\times 100$$

where *X* is the lipoxygenase activity (%), ΔAbs234 nm/min sample is the enhancement in absorbance at 234 nm per minute per mg of the sample lipoxygenase tubes under trial conditions, and ΔAbs234 nm/min control is the enhancement in absorbance at 234 nm per minute per mg of the control lipoxygenase tubes under trial conditions.

### 2.8 Preparation of the solutions for drying process

After fermentation, the samples were centrifuged at 12,000 *g* for 15 min at 4°C using a refrigerated centrifuge (Jouan, France), then 3 wt.% of Tween 20 was also added in order to maintain the stability during the drying process. One milliliter was used to count the viable bacteria in the solution before the drying process as the initial count and the residue was divided into two portions: one for electrospray drying and the other for freeze‐drying.

### 2.9 Physical properties of the solutions

The Wilhelmy plate method was applied to determine the surface tension by a tensiometer (Kruss K100 Tensiometer). The electrical conductivity of the fermented solutions was determined using a conductometer (Jenway model 4510). Programmable viscometer (Brookfield DV3T) using spindle no. SC4‐18 was applied for viscometric assessment. All experiments were made in triplicate at 25°C and the data were expressed as mean ± standard deviation.

### 2.10 Electrospray‐assisted drying

Electrospraying was conducted in a horizontal mode equipment (Fanavaran Nano‐Meghyas, Iran) with a variable high voltage 0–30 kV power supply. Solutions were injected by a 1 ml sterile plastic syringe under steady flow rate of 0.1–1 ml h^−1^. The voltage used varied between the range of 10 and 20 kV, and the applied distance of the needle tip to collector was different from 10 to 15 cm. These parameters were applied precisely to obtain the optimum condition.

### 2.11 Freeze‐drying

Frozen samples were freeze‐dried in a freeze‐drier (Dena Vacuum Industry, Co., Ltd.) at a condenser temperature of −80°C and chamber pressure of 0.02 mbar for 24 h.

### 2.12 Scanning electron microscopy (SEM)

The morphological aspects of the freeze‐dried and electrosprayed powder were assessed by a TESCAN VEGA‐LMU scanning electron microscope (Czech Republic) at an accelerating voltage of 15 kV and a working distance of 6–7 mm. Prepared samples on aluminum foils were coated directly by a thin layer of gold–palladium mixture under vacuum before their morphology was determined using SEM.

### 2.13 Viability of the probiotics

To investigate the effect of freeze‐dry and electrospray processes on initial cell counts of the probiotic bacteria and to compare the viability of the probiotics during the storage at 25°C after being processed by both methods, plate count agar was used at intervals of 7 days for 70 days. For this purpose, 0.01 g of dried powder samples was added to 9 ml sterilized deionized water and serial dilutions were made, then after incubation on MRS agar at optimal conditions for 72 h, the logarithm of the number of colony‐forming units per gram (log CFU/g) was calculated.

### 2.14 Statistical analysis

All of the measurements were done in triplicate. Mean and standard deviation values were computed applying Microsoft Excel 2016. A one‐way analysis of variance (ANOVA), followed by Tukey's test, was used to specify statistical differences between the mean values of each test. A significance level of *p* < .05 was applied throughout the work.

## 3 RESULTS AND DISCUSSION

### 3.1 pH

**FIGURE 1:**
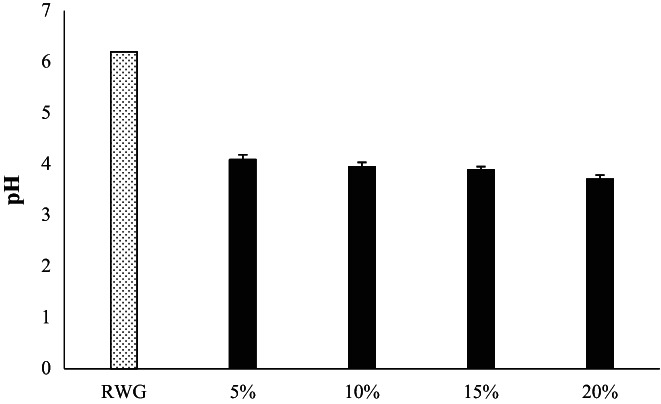
Effect of mixed fermentation by *Lactobacillus acidophilus* and *Lactobacillus plantarum* on the pH level of different concentrations of WG (5%, 10%, 15%, and 20%)

The alterations in pH value in 5%, 10%, 15%, and 20% suspensions of WG after 24 h fermentation by coculture of *L. acidophilus* and *L. plantarum* are represented in Figure 1. The initial pH of crude and fresh WG was 6.2 and after mixed fermentation, the pH value of different samples ranges from 4.09 to 3.71. According to Figure 1, it has been shown that the acidification rate was highest in the FWG with concentration of 20% and lowest in 5% FWG. As a result, with increasing WG concentration, the bacteria were more active. The pH level can be considered as an indicator of the activity and growth of lactic acid bacteria, which ferment carbohydrate substrates to produce lactic acid as the main product, and pH reduction following lactic acid production is the primary effect of lactic acid fermentation by lactic acid bacteria. These results were in agreement with Rizzello et al. (2010) that the activity of the lactic acid bacteria on WG substrate was very remarkable and prebiotic compounds released during fermentation had a positive effect on increasing the growth of bacteria (Rizzello et al., 2010; Seleet et al., 2016).

### 3.2 Effect of fermentation on lipase and lipoxygenase activity

**FIGURE 2:**
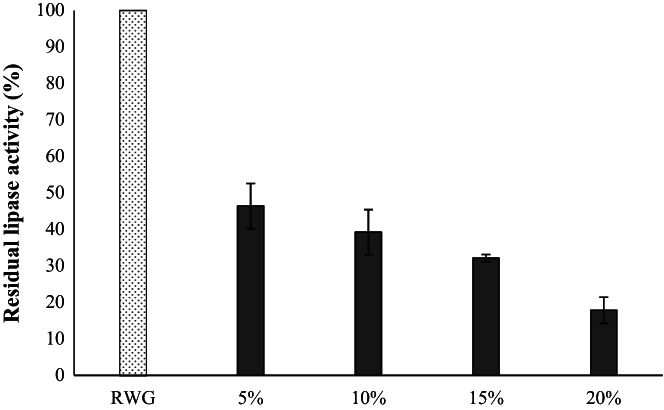
Effect of mixed fermentation by *Lactobacillus acidophilus* and *Lactobacillus plantarum* on the lipase activity of different concentrations of WG (5%, 10%, 15%, and 20%)

Figure 2 summarizes the level of residual activity of WG lipase at four different fermentation conditions. As can be seen in the figure, after fermentation, a notable reduction in the enzyme activity level is observed (53.58% – up to a maximum of 82.15%) in all samples, where the reduction level was higher in the more concentrated suspensions. Thus, the 20% WG suspension was ranked first. The enzymes have an optimum pH for their activity, and outside of that pH, by changing the structure of the active site, its affinity to the substrate is decreased, thereby the activity of the enzyme is reduced.

Lipases and lipoxygenase of WG have an optimum pH of ca. 7.0–8.0 and ca. 4.0–10.0, respectively, and the activity decrease outside of these pH ranges (Boukid et al., 2018). Lactic acid fermentation decreases the activity of these destructive endogenous enzymes by acid production and pH reduction.

**FIGURE 3:**
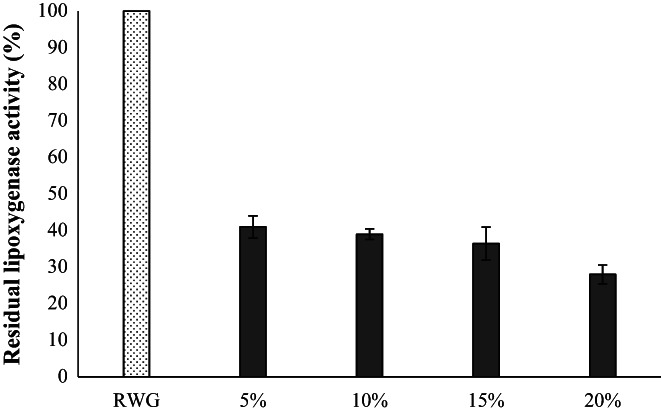
Effect of mixed fermentation by *Lactobacillus acidophilus* and *Lactobacillus plantarum* on the lipoxygenase activity of different concentrations of WG (5%, 10%, 15%, and 20%)

Figure 3 presents the residual lipoxygenase activity in this study. It illustrates that the trend of changes in lipoxygenase activity by different concentrations is similar to that of lipase, except that the final values are different (59% reduction for suspension with 5% WG and 72% reduction for suspension with 20% WG), which is probably due to the final pH value in different samples and the degree of pH variation from the optimal activity pH for each enzyme.

In earlier studies, *L. plantarum* LB1 and *L. rossiae* LB5 were applied as starters to produce sourdough fermented wheat germ (SFWG), and it was found that lipase activity in SFWG was ca. 2.6‐fold less than that found in raw WG (Rizzello et al., 2010). Comparison of the effect of toasting and sourdough fermentation on inactivation of WG lipase and lipoxygenase indicated that both of these methods were remarkably effective in decreasing the activity of enzymes and sourdough fermentation can be an appropriate alternative to toasting with a 60.38 reduction in lipoxygenase activity. Also, applying of fermentation and subsequent toasting could decrease lipoxygenase activity by 79.25% (Marti et al., 2014).

### 3.3 Physical properties of the solution

Physical properties of different solutions are represented in Table 1. The surface tension values due to the addition of surfactant in all samples were low ranging from 26.97 ± 0.06 to 39.64 ± 0.04. Surfactant by reduction in the surface tension could improve the electrospray process. As can be seen in the table, by increasing the WG concentration in fermented samples, electrical conductivity was increased from 3222.33 ± 2.51 in 5% concentration to 5126 ± 2.64 in 20% concentration that was probably due to the more activity of bacteria because they can convert low‐conductivity substrates into high‐conductivity substrates. Among the samples as can be predicted, the highest viscosity value related to the sample that is more concentrated (20% FWG). Previous researchers proposed that higher viscosities improve the process yields (Zaeim et al., 2018).

**TABLE 1:** Physical properties of electrosprayed solutions^a^

Matrix	Surface tension (mN/m)	Conductivity (μS/cm)	Viscosity (mPa s)	Stable jet formation
5% w/v FWG	39.64 ± 0.04	3223.33 ± 2.51	103.03 ± 2.43	No
10% w/v FWG	34.17 ± 0.06	3577 ± 1.73	290.66 ± 3.05	No
15% w/v FWG	30.79 ± 0.03	4244 ± 3.6	366 ± 4.58	Yes
20% w/v FWG	26.97 ± 0.06	5126 ± 2.64	523.33 ± 3.51	Yes

^a^ All values are mean ± standard deviation of three replicates.

Effect of polymeric solution characteristics on electrospray and electrospinning processes have been studied by different researchers (Librán et al., 2017; Zaeim et al., 2018) and the obtained results revealed that many solutions with different surface tension, conductivity, and viscosity have well electrosprayability, which defined that for electrospraying a solution, the combination effect of these three parameters should be considered (Librán et al., 2017; Rostamabadi et al., 2021).

### 3.4 Morphology of the particles

**FIGURE 4:**
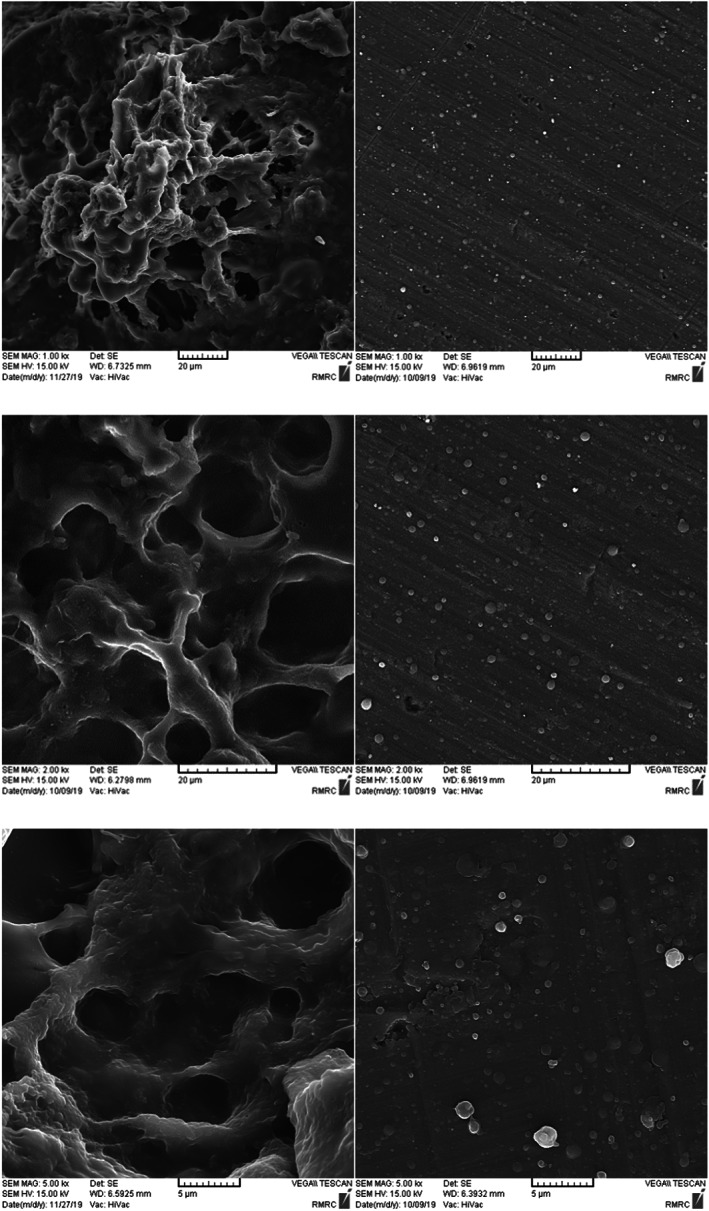
SEM images of freeze‐dried powder (left column) and electrosprayed powder (right column) based on FWG

The SEM images of the produced powders by electrospray and freeze‐drying methods are presented in Figure 4.

Nonparticular shape and porous structure in freeze‐dried powders can be observed (Figure 4 left column). Rajam et al. (2012) reported that freeze‐dried microcapsules were irregular in shape and have spongy structure.

Some key factors affect the electrospraying process. Other than physical properties of the solution (surface tension, electrical conductivity, and viscosity) as specified earlier, some instrumental factors such as applied voltage, flow rate, needle gauge, and tip‐to‐collector distance should be optimized for every under trial solution in order to obtain fine droplets with high productivity (Ma et al., 2021; Mendes & Chronakis, 2021; Ta et al., 2021; Tanhaei et al., 2021; Zaeim et al., 2018), though this is a trial‐and‐error method and should be considered separately for each solution. In this work, electrospraying of the solutions was performed at a range of applying voltage within 10–20 kV, feed flow rate of 0.1–1 ml h^−1^ and tip‐to collector distance of 10–15 cm. FWG (5% and 10% solutions) was not very suitable for electrospraying may be due to the high amount of water, thus only water drops were collected on the collector and the produced powder was rarely visible. FWG (15% solution) could be electrospray by optimizing the instrument factors especially higher than 16 kV, but the productivity was very low and the water droplets also were seen on the collector. For 20% solution of FWG, powder was achieved by different instrument parameters, but the most productive condition with the minimum water droplets was 18 kV applying voltage at 0.3 flow rate with 12 cm distance between tip to collector that microcapsules are shown in Figure 4 right column. Microencapsulates made by electrospray drying method are in semiglobular shape and almost are uniform. Figure 4 clearly shows that the capsules are well formed and there is no evidence of agglomeration and coagulation, indicating that the sample has good electrosprayability and the instrument parameters are well adjusted, thus the solvent is almost completely evaporated. Previous authors reported that spherical and uniform particles can be produced by electrospraying process (Gomez‐Mascaraque et al., 2016; Librán et al., 2017; Moayyedi et al., 2018; Zaeim et al., 2018).

### 3.5 Viability of microencapsulated bacteria after drying process and during the storage

**FIGURE 5:**
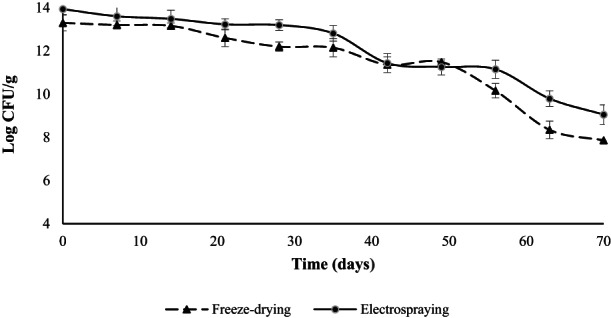
Viability of probiotics in freeze‐dried and electrosprayed FWG powder during the storage at 25°C

Viability of probiotics after electrospray drying was specified and compared to freeze‐drying. Figure 5 summarizes the results from viability loss of probiotics during the storage after freeze‐drying and electrospraying process. The number of viable bacteria before the drying process was counted as 14.48 ± 0.2 log cfu/g of dry matter. From Figure 5, it can be achieved that the freeze‐drying process reduced the initial amount of viable bacteria by around 1.2 log cfu/g. In freeze‐drying process, cell losing mostly occurs during freezing step (Rajam & Subramanian, 2022). Crystal formation during freezing can cause damage to the cell membrane and consequently their death (José et al., 2015; Rajam & Subramanian, 2022). After drying, the number of viable bacteria was almost stable at the first 14 days of storage and no significant reduction was observed. Then, they showed a downward trend in the following days of storage and the number of viable bacteria reached 7.86 ± 0.03 log cfu/g on the last day. Freeze‐drying has always been suggested as an effective way of preserving the viability of probiotics (José et al., 2015; Librán et al., 2017; Moayyedi et al., 2018). Most researches focused on survival during the process of freeze‐drying, not during storage period and exposure in different environmental condition (Rajam & Subramanian, 2022). About 1.3 log cycle reduction in viability of freeze dried *L. plantarum* NCIMB 8826 was observed (Albadran et al., 2015). In other study by Zaeim et al. (2018), *L. plantarum* was encapsulated by freeze‐drying using 35% acacia gum solution and after drying processes approximately 1 log cycle reduction in its viability was reported. Microencapsulated *L. plantarum* NRRL B4496 in the enzymatically extracted purple rice bran fiber showed less than 1 log decline after freeze‐drying, whereas unencapsulated cells had more than 6 log reductions.

As a result of the electrospraying process, 0.55 log cfu/g decrease in the number of viable bacteria from initial count occurred. Then, a slight reduction in the viable bacteria was observed during the first 35 days of storage and finally the viability of encapsulated cells decreased from 13.93 ± 0.25 log cfu/g in the first day to 9.05 ± 0.45 log cfu/g in the last day of storage. In a study, electrospinning was applied for encapsulating *Bifidobacterium* in whey protein concentrate and pullulan and no remarkable decrease in viability was observed (López‐Rubio et al., 2012). Results from another study represented 4% reduction in viability of *Bifidobacterium longum* after freeze‐drying from initial count, while this value was constant after electrospraying (Librán et al., 2017). Zaeim et al. (2018) reported that electrospraying had no significant effect on the viability of the *L. plantarum* and about 96% passed through the process alive. It has been confirmed that electrospray method does not affect bacterial count (Librán et al., 2017). Other study reported that the probiotics viability loss was less than 1 log cfu/g during electrospraying (Gomez‐Mascaraque et al., 2016). According to the results, the electrospraying method was more capable of maintaining the viability of the probiotic bacteria. Previously authors confirmed the better performance of electrospray processing in comparison with traditional methods such as spray‐drying and freeze‐drying (Gomez‐Mascaraque et al., 2016; Librán et al., 2017; Zaeim et al., 2018). The superiorities of electrohydrodynamic atomization for probiotics over other techniques, such as spray‐drying and freeze‐drying, including lower viability loss, better productivity performance at ambient temperature, avoiding the use of organic solvents, producing more uniform capsules (electrospraying) or fibers (electrospinning) regarding morphology, and continuous usability would allow to scale up the process (Mendes & Chronakis, 2021). Electrohydrodynamic atomization show promise as novel encapsulation method for probiotics, though more detailed researches are needed in this field.

## 4 CONCLUSIONS

In conclusion, a novel nondairy‐fermented probiotic powder based on stabilized WG using mixed fermentation and electrospraying as a novel encapsulation method have been developed. At first, in order to enhance the stability of WG, the fermentation effect by coculture of *L. acidophilus* and *L. plantarum* on its lipase and lipoxygenase activity was evaluated. Then, the preparation of the solutions for drying process and investigation of physical properties (surface tension, electrical conductivity, and viscosity) of them was done and the electrosprayability of samples was evaluated at different conditions. The results disclosed that 20% solution of FWG has good electrosprayability especially at 18 kV applying voltage, 0.3 flow rate, and 12 cm distance between tip to collector. Morphological assessments also showed that semiuniform particles could be obtained by electrospray process, while freeze‐dried powder had nonparticular shape and porous structure. Finally, viability studies showed about 1.2 log cfu/g and 0.55 log cfu/g decrease in the number of viable bacteria from initial count (14.48 ± 0.2 log cfu/g) as a result of freeze‐drying and electrospraying, respectively. Furthermore, 7.86 ± 0.03 log cfu/g in freeze‐dried and 9.05 ± 0.45 log cfu/g in electrosprayed samples survived after 70 days of storage at 25°C.

Our findings propose mixed fermentation by *L. acidophilus* and *L. plantarum* as a promising tool for stabilizing the WG, and also consider the electrospraying as an effective method for encapsulation of *L. acidophilus* and *L. plantarum*. Considering the importance of nondairy probiotic products, the viability of probiotics during the storage as well as the necessity of optimal use of WG as a low‐cost nutrient by‐product, the conducted research is important from various aspects of biotechnology, health, and economy.

## CONFLICT OF INTEREST

The authors declare no conflict of interest.

## Data Availability

The datasets generated during and/or analyzed during the current study are available from the corresponding author on reasonable request.
